# Duck Tembusu virus infection induces mitochondrial-mediated and death receptor-mediated apoptosis in duck embryo fibroblasts

**DOI:** 10.1186/s13567-022-01070-9

**Published:** 2022-07-07

**Authors:** Yuhong Pan, Wenjun Cai, Anchun Cheng, Mingshu Wang, Shun Chen, Juan Huang, Qiao Yang, Ying Wu, Di Sun, Sai Mao, Dekang Zhu, Mafeng Liu, Xinxin Zhao, Shaqiu Zhang, Qun Gao, Xumin Ou, Bin Tian, Zhongqiong Yin, Renyong Jia

**Affiliations:** 1 Research Center of Avian Disease, College of Veterinary Medicine, Sichuan Agricultural University, Chengdu, 611130 Sichuan China; 2 Institute of Preventive Veterinary Medicine, Sichuan Agricultural University, Chengdu, 611130 Sichuan China; 3 Key Laboratory of Animal Disease and Human Health of Sichuan Province, Chengdu, 611130 Sichuan China

**Keywords:** DTMUV, apoptosis, DEF, mitochondrial apoptotic pathway

## Abstract

Duck Tembusu virus (DTMUV) is a pathogenic flavivirus that has caused enormous economic losses in Southeast Asia. Our previous study showed that DTMUV could induce duck embryo fibroblast (DEF) apoptosis, but the specific mechanism was not clear. In this study, we confirmed that DTMUV could induce the apoptosis of DEFs by DAPI staining and TUNEL staining. Furthermore, we found that the expression levels of cleaved-caspase-3/7/8/9 were significantly upregulated after DTMUV infection. After treatment of cells with an inhibitor of caspase-8 or caspase-9, DTMUV-induced apoptosis rates were significantly decreased, indicating that the caspase-8-mediated death receptor apoptotic pathway and caspase-9-mediated mitochondrial apoptotic pathway were involved in DTMUV-induced apoptosis. Moreover, we found that DTMUV infection not only caused the release of mitochondrial cytochrome C (Cyt C) and the downregulation of the apoptosis-inhibiting protein Bcl-2 but also reduced the mitochondrial membrane potential (MMP) and the accumulation of intracellular reactive oxygen species (ROS). Key genes in the mitochondrial apoptotic pathway and death receptor apoptotic pathway were upregulated to varying degrees, indicating the activation of the mitochondrial apoptosis pathway and death receptor apoptosis pathway. In conclusion, this study clarifies the molecular mechanism of DTMUV-induced apoptosis and provides a theoretical basis for revealing the pathogenic mechanism of DTMUV infection.

## Introduction

DTMUV is an arbovirus of the genus *Flavivirus*, family *Flaviviridae* [1]. DTMUV is an enveloped virus with a positive-polarity RNA genome that is approximately 11 kb in length [2]. Beginning in April 2010, an outbreak of DTMUV occurred in major duck farming regions in China [3]. Since then, DTMUV has also caused serious harm to the duck industries in Thailand, Malaysia, and other Southeast Asian countries as well as China [4–6]. Slow growth of poultry, sharp declines in egg production, and even suspension of production have caused major economic losses [7]. Several studies have revealed that DTMUV has a wide host range, infecting ducks, chickens, geese, pigeons and house sparrows [8, 9]. Moreover, more than 70% of duck industry workers have been reported to have antibodies against DTMUV in the serum samples tested, and ~50% of oral swab samples have been found to be positive for DTMUV RNA [10]. These findings indicate that DTMUV is likely to spread from ducks to other nonavian hosts and even humans. Therefore, it is necessary to explore the pathogenic mechanisms of DTMUV to enable the design of better disease control strategies.

Apoptosis, also known as programmed cell death (PCD), is a self-protective mechanism used by multicellular organisms to clear senescent, damaged or pathogen-infected cells [11]. Apoptotic cells are characterized by cell shrinkage, chromatin aggregation, DNA fragmentation, mitochondrial swelling, and ultimately the formation of apoptotic bodies containing nuclear or cytoplasmic debris surrounded by cell membranes and cleared by phagocytes [12].

Apoptosis has been shown to be induced by two classical pathways: the intrinsic and extrinsic pathways [13]. The chief players in the intrinsic pathway are mitochondria, the key energy producers of the cell [14]. The triggers for this apoptotic pathway are numerous and include DNA damage, oxidative damage, and endoplasmic reticulum stress (ERS) [15]. Infection with pathogens can also trigger intrinsic apoptosis through the aforementioned stresses [16]. These stimuli lead to the activation of Bax, which is mediated by BH3-only Bcl-2 family members such as Bim and PUMA [17, 18]. Bax activation manifests as oligomerization at the mitochondria, leading to the release of apoptotic proteins such as Cyt C [19] and endonuclease G (EndoG) [20]. Ultimately, this stimulates the formation of a multimeric complex composed of Apaf-1, Cyt C and procaspase-9, which is known as the apoptosome [21].

The extrinsic pathway is triggered through activation of extracellular death receptors of the tumour necrosis factor receptor (TNFR) superfamily, which bind specific ligands [22]. This family of receptors includes TNFR, Fas, death receptor 4 (DR4) and DR5 [23]. Upon activation of these receptors, initiator caspases such as pro-caspase-8 and pro-caspase-10 are recruited to the death receptor, where they become activated [24]. Critical to the formation of such complexes are the Fas-associated death domain (FADD) and TNF receptor-associated death domain (TRADD) proteins [25, 26]. These proteins act as adaptors that bridge death receptors and caspases. The complex of receptors, adaptors and caspases is typically referred to as the death-induced signalling complex (DISC) [27]. The formation of this complex leads to cleavage and activation of effector caspases such as caspase-3/7, whose protease activity directly acts on cellular substrates that are critical for the integrity of the cells [28]. Breakdown of these structural components leads to the hallmarks of apoptotic cell death, including nuclear DNA fragmentation, destruction of the cytoskeleton, blebbing and packing of cellular contents into small membrane-bound vesicles known as apoptotic bodies [29].

Apoptosis is a key innate defence mechanism that inhibits virus replication and clears virus-infected cells, which is important for maintaining healthy development of the body and normal function of the immune system [30]. However, many viruses have evolved strategies to prevent or delay apoptosis during viral replication until sufficient progeny viruses are produced [31]. In addition, some virus-induced apoptosis can also enhance viral spread, leading to tissue damage and disease. It has been found that a variety of flaviviruses, such as JEV, DENV, ZIKV, and WNV, can lead to apoptosis of host cells [32–35], and DTMUV, a new flavivirus, has similar biological properties. To address the uncertainties, this study preliminarily explored the molecular mechanism of induction of host cell apoptosis during DTMUV infection, providing a theoretical basis for revealing the pathogenic mechanism of DTMUV infection.

## Materials and methods

### Cells and viruses

DEFs were obtained from 10-day-old duck embryos according to the manufacturer’s instructions [36]. The cells were grown in medium supplemented with 10% newborn bovine serum (NBS) (Gibco, Gaithersburg, MD, USA) and maintained in Dulbecco’s modified Eagle’s medium (DMEM) (Gibco Life Technologies, Shanghai, China) at 37 °C in a humidified atmosphere of 95% air and 5% CO_2_. When the DEFs reached  90% confluence, they were mock-infected or infected with the DTMUV CQW1 strain (GenBank accession number KM233707.1) at a multiplicity of infection (MOI) of 1 [37, 38]. After the virus was adsorbed in a 37 °C, 5% CO_2_ incubator for 1 h, the virus medium was replaced with maintenance medium (DMEM containing 2% NBS), and the cell samples were collected at the indicated time points. Each group was processed with three independent biological replicates.

### DAPI staining of the cell nucleus

4’,6-Diamidino-2-phenylindole (DAPI) staining of the nucleus was conducted using a standard procedure. Briefly, DTMUV-infected and control cells were collected and fixed with 4% paraformaldehyde (PFA) for 1 h. Subsequently, the cells were permeabilized with 0.1% Triton X-100 for 30 min and stained with DAPI for 10 min. The nuclei were observed with a fluorescence microscope.

### Terminal deoxynucleotidyl transferase-mediated dUTP nick end-labelling (TUNEL) assay

TUNEL staining was used to detect apoptosis-specific nuclear DNA fragmentation. Briefly, cells were fixed in 4% PFA for 30 min at room temperature, permeabilized for 5 min and washed with PBS three times. Then, the cells were stained using a TUNEL kit (Beyotime, China) according to the manufacturer’s instructions. The slides were covered and incubated at 37 ℃ for 1 h in the dark. Finally, anti-fluorescein quenching solution was added. TUNEL-positive cells were visualized by green fluorescence, and images were observed under a fluorescence microscope.

### Cell viability assays

Cell viability was measured using an MTT assay according to the manufacturer’s instructions. Briefly, the cells were seeded in 96-well plates, and cell viability was detected at 36 h after IETD-FMK, Z-LEHD-FMK, and Z-VAD-FMK treatment.

### Flow cytometric analysis of apoptosis

Apoptotic cells were detected by flow cytometry (FCM). The cells were washed twice with cold PBS and then resuspended in 1 × binding buffer at a concentration of 1 × 10^6^ cells/mL. Then, 100 µL of the solution (1 × 10^5^ cells) was transferred to a 5 mL culture tube. Five microlitres of Annexin V-fluorescein isothiocyanate (V-FITC) (BD Pharmingen, USA) and 5 µL of propidium iodide (PI) (BD Pharmingen, USA) were added to 100 µL of cell suspension and incubated at RT (25 °C) in the dark for 15 min. Then, 1 × binding buffer (400 µL) (BD Pharmingen, USA) was added to the mixture, and the percentage of apoptotic cells was assayed by FCM within 1 h.

### Determination of MMP

Changes in cellular MMP were determined using a JC-10 staining kit (Sigma, USA). DEFs were seeded at a density of 2 × 10^6^ cells in 6-well culture plates. After the adherent cells grew, they were infected with DTMUV or mock-infected for 12, 24, 36, 48 and 60 h at 37 °C in a CO_2_ incubator. We prepared the JC-10 dye loading solution by adding 25 μL of 200 × JC-10 to 5 mL of assay buffer. Then, adherent cells were detached with trypsin, collected by centrifugation, resuspended in 500 μL of the JC-10 dye loading solution and incubated at 37 °C in a 5% CO_2_ incubator for 30 min protected from light. The stained cells were then collected by centrifugation and washed with assay buffer. After washing, the cell suspension was centrifuged once more and then resuspended in 1 mL assay buffer. Then, the cells were observed, and the data were collected with a fluorescence microscope and a multifunctional microplate reader. When the MMP is high, JC-10 concentrates in the mitochondrial matrix, forming aggregates and generating red fluorescence. In apoptotic cells, MMP collapse results in the failure to retain JC-10 in the mitochondria; therefore, JC-10 becomes a monomer, producing green fluorescence. The relative ratio of red to green fluorescence was used to represent MMP.

### Determination of intracellular ROS levels

Detection of intracellular ROS in DEFs was performed with a fluorometric intracellular ROS assay kit (Sigma–Aldrich, USA) based on a superoxide fluorescent probe (dihydroethidium). DEFs were plated in 96-well black microplates with clear flat bottoms to a cellular concentration of 1 × 10^4^ cells/mL. The cells were then allowed to adhere for 24 h and infected with DTMUV, and the cells were harvested according to the instructions at 6, 12, 24, 36, 48 and 60 h of infection. The ROS reagent was reconstituted with dimethyl sulfoxide and then diluted in the assay buffer. Shortly afterwards, the ROS reagent solution was added to each well, and the plates were incubated for 1 h. Subsequently, the data were collected using a fluorescence microscope, and the fluorescence intensity was measured in a multifunctional microplate reader (Thermo Scientific, Massachusetts, USA) with an excitation wavelength of 520 nm and an emission wavelength of 605 nm. The data are shown as relative fluorescence compared to the fluorescence of the negative control.

### Cytosolic fractionation

To detect the release of mitochondrial proteins, cell pellets were washed with cold PBS. Mitochondria were isolated using a Cell Mitochondria Isolation Kit (Beyotime, China). Briefly, cells were resuspended in lysis buffer and passed 10 times through a 26 G needle. The cell lysates were centrifuged, and both the supernatants and precipitates were collected. The protein concentrations of the cytosolic fraction (supernatant) and mitochondrial fraction (pellet) were determined with a BCA Protein Assay Kit (Beyotime, China). The protein samples were separated by SDS–PAGE for Western blotting.

### Western blot analyses

Cells were cultured in 6-well plates and harvested with lysis buffer (Thermo Fisher Scientific, USA) containing a mixture of protease and phosphatase inhibitors. Equal amounts of the samples were then separated by SDS–PAGE (12% polyacrylamide), transferred to polyvinylidene difluoride membranes, and incubated with primary and secondary antibodies (rabbit antibodies against pro-caspase-3/7/8/9 were purchased from ABclonal; antibodies against cleaved-caspase-3/7/8/9 were purchased from CST; a DTMUV-E mouse monoclonal antibody was prepared by our laboratory; antibodies against β-actin, PARP, Cyt C, VDAC1 were purchased from Proteintech; rabbit antibodies against Bcl-2 were purchased from Beyotime; and HRP-conjugated goat anti-mouse IgG and HRP-conjugated goat anti-rabbit IgG were purchased from Proteintech and visualized using an ECL system (Bio-Rad Laboratories)). The expression of β-actin, used as a loading control, was detected with an anti-β-actin mouse monoclonal antibody (Proteintech, China).

Total RNA was isolated from DTMUV-infected and mock-infected cells at different time points using TRIzol reagent (Invitrogen, CA, USA). The purity of all RNA samples was detected by analysing the A260/A280 ratio using a NanoDrop ND-1000 spectrophotometer (NanoDrop Technologies), which was expected to be 1.8–2.0. First-strand cDNA was obtained via reverse transcription of extracted RNA with a PrimeScript™ RT Reagent Kit (TAKARA). Q–RT–PCR was performed using a SYBR Green real-time PCR assay (CFX96 Bio-Rad, Hercules, CA, USA). The Q–RT–PCR was set up in a total volume of 20 µL containing 2 µL of cDNA, 10 µL of SYBR Premix (Tli RNaseH Plus), 1 µL of forward/reverse primer and 6 µL of ddH_2_O. The duck β-actin gene was used as an internal control gene to normalize the targeted gene expression value. The quantity of mRNA was calculated by the 2^−ΔΔCt^ method and is presented as the mean ± SEM (*n* = 3). The relative gene expression was calculated using the mean values obtained with the arithmetic formula ∆∆Ct. The primers employed are listed in Table 1.

**Table 1:** **Primers for Q–RT–PCR analysis of gene expression**.

Target gene	Forward (5'-3')	Reverse (5'-3')
AIF	AGGCTGACACTCTTCCG	TCTGTGGTCCAGTTGCTC
XIAP	AGGCACTGACTATGACC	TCACTTTACAGCCTTCG
Apaf-1	AGAGGGCACAAGGAAGCTATCA	AACTTACTACCATCAGGCGAAACA
Smac	AGTCTGGCAGGTGGTG	CGTAACGCTGTCATCC
Bak	TCACCAAGGAGAATGCCTACGA	GCCTGTTATGCCGTGCTGGTA
Bcl-xL	AAAATGTCCAGCGGCAACC	AAGCCAACTCAGTCCTGTTCTCA
PARP	CTGGACTAAGTGCGTTGC	AGCCTCTGGAGGGAATA
Bid	CTGTCGCAGGCTGTGGAAGT	AGACACGCTGGAGAAGGAAGG
Cyt C	TGAGGATACCCTGATGGAGTACTTG	CTTCGCAGTGGCATCTTTCAG
FasL	AGCAGCCAGATGTTGAGCCA	GTCCCTCTCCCTGAGCTTGAATA
Fas	TATCTGCACTGACTTCAAGCGTATT	TGTTGGCTGTTGCATGACTGG
TNFSF10	CCCTGACACTTCCACG	CGATTCCCAGTTGCTTA
TNFRSF10B β-actin	TGCGAGATGTGCCAGAAGT GATCACAGCCCTGGCACC	GGCGAAGACGTGGAAAGA CGGATTCATCATACTCCTGCTT

### Statistical analysis

All experiments were performed in triplicate, and the data analysis was performed using GraphPad Prism 7.0. The results are expressed as the mean ± SEM, and statistical significance was assessed with Student’s *t* test. *p* values less than 0.05 were considered to indicate statistical significance.

## Results

### DTMUV induces apoptosis in DEFs

**Figure 1:**
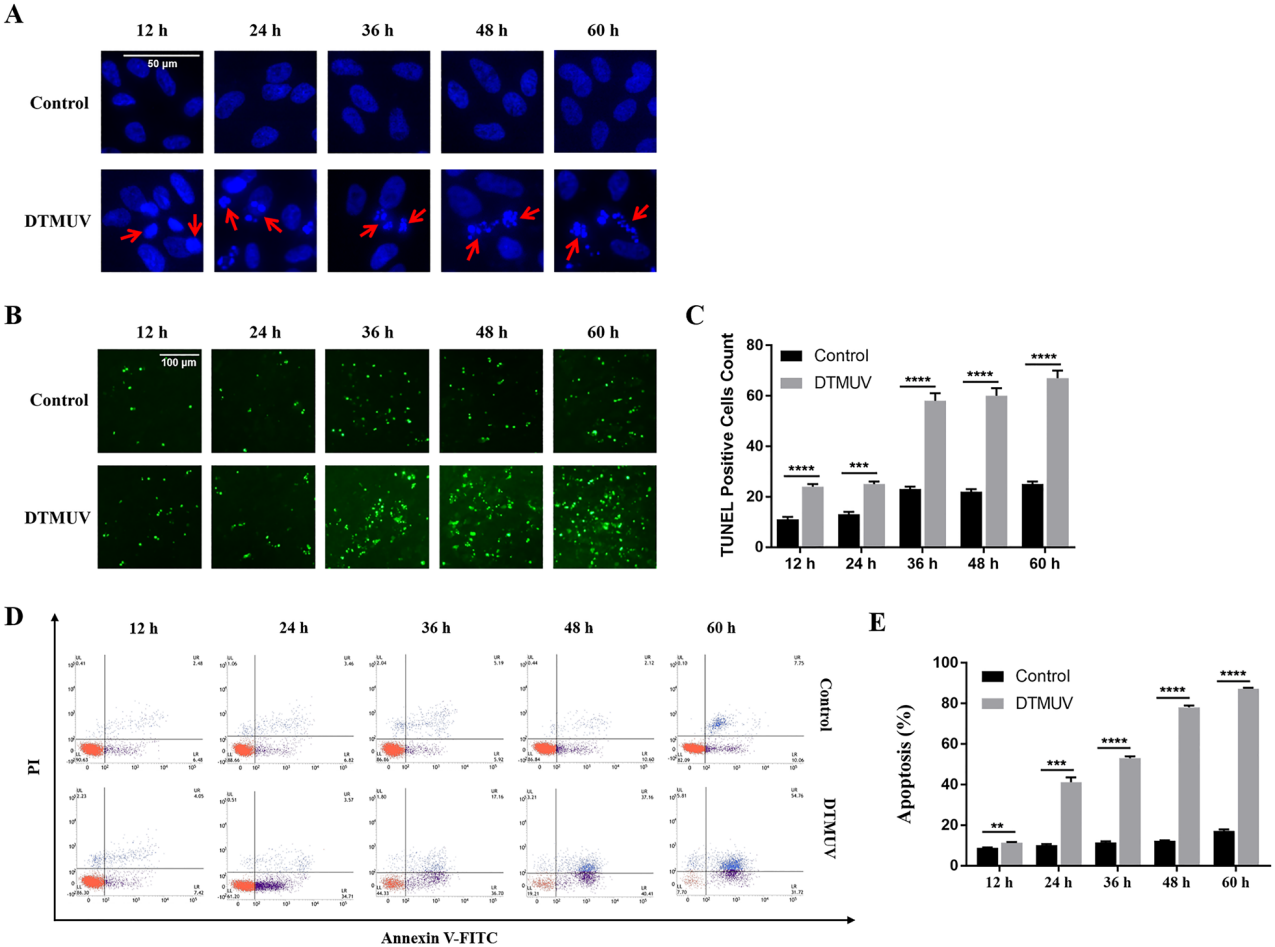
**DTMUV induces apoptosis in DEFs. A** Nuclear morphological changes in cells infected with DTMUV for the indicated number of hours. At 24, 36, 48, and 60 h, the arrows indicate that the nuclei of infected cells appeared as typical fragmented and marginated apoptotic bodies. **B** Representative photographs of TUNEL staining in DEFs infected with DTMUV for the indicated number of hours. **C** Quantitative analysis of TUNEL-positive cell content among groups. All values are denoted as the means ± SEMs for green cells from 10 images obtained in randomly selected fields from each group. **D** Ratio of apoptosis as analysed by double staining with annexin V-FITC and PI followed by flow cytometry. Representative images are shown (*n* = 3). **E** Bar graph of the percentage of apoptotic cells. Statistical significance was assessed with Student’s *t* test, **p < 0.01, ***p < 0.001, ****p < 0.0001.

DAPI staining was performed to observe the morphological changes in the cell nucleus (Figure 1A). The contraction of the nucleus and the aggregation of chromatin were observed at 12 and 24 h in the DTMUV-infected cells, which are denoted by arrowheads. In addition, DAPI staining at 24, 36, 48, and 60 h revealed the presence of apoptosis-associated morphological changes, such as nuclear fragmentation and apoptotic body formation.

When cells undergo apoptosis, some DNA endonucleases are activated, and these endonucleases cut off the genomic DNA between nucleosomes. When genomic DNA is broken, the exposed 3'-OH can be catalysed by terminal deoxynucleotidyl transferase (TdT) to add FITC-labelled dUTP (fluorescein-dUTP), which can be analysed by fluorescence microscopy. This is the principle of the TUNEL method to detect apoptosis. Compared with that in the control group, the green fluorescence in the group of DEFs infected with DTMUV increased significantly from 36 h, and the number of TUNEL-positive cells was increased after DTMUV infection, indicating that DTMUV infection can trigger the fragmentation of intracellular DNA and induce apoptosis (Figures 1B and C). Flow cytometric analysis was performed after staining with Annexin V-FITC and PI, and the results showed that DTMUV had a significant proapoptotic effect that was obviously time-dependent (Figures 1D and E).

### Caspases are involved in DTMUV-induced apoptosis in DEFs

**Figure 2:**
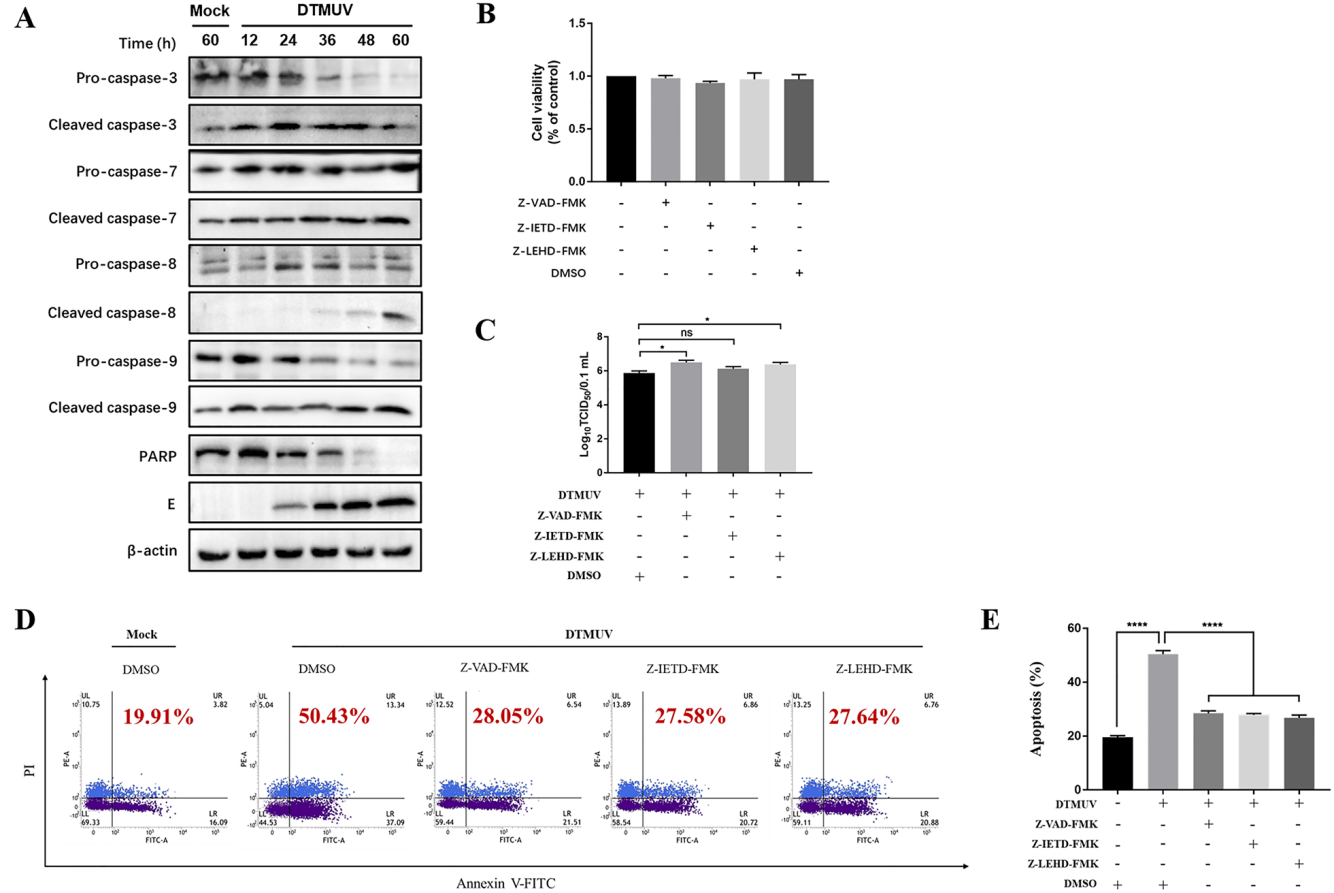
**Caspases are involved in DTMUV-induced apoptosis in DEFs. A** Effects of DTMUV on caspases and PARP in infected DEFs. **B** Changes in DEF viability at 36 h following treatment with Z-VAD-FMK, Z-IETD-FMK and Z-LEHD-FMK. **C** DEFs were pretreated with inhibitors for 2 h and then infected with DTMUV for 60 h. After incubation, the viruses were collected, and the viral titres were determined. The titres are presented as the Log_10_TCID_50_/0.1 mL. **D** Changes in apoptosis of DEFs induced by DTMUV following treatment with Z-VAD-FMK, Z-IETD-FMK and Z-LEHD-FMK. **E** Bar graph of the percentage of apoptosis in Panel **D**; *p < 0.05, ****p < 0.0001.

Activation of caspases is essential during apoptosis. It is well known that mitochondria-mediated and death receptor-mediated pathways induce apoptosis via caspase-9 and caspase-8, respectively, and the apoptosis effector molecule is caspase-3/7. To determine the molecular mechanism of DTMUV-induced apoptosis, we first explored the roles of the caspases in the process of apoptosis and analysed the pathway through which DTMUV induced apoptosis. The DTMUV-E protein was used to indicate the virus infection process. The results are shown in Figure 2A. DTMUV infection promoted increased expression of cleaved caspase-3, caspase-7, caspase-8 and caspase-9. In addition, the DNA repair enzyme PARP (poly(ADP-ribose) polymerase), which serves as a substrate for cleaved caspase-3/7, was inactivated by cleavage.

To further determine the roles of caspases in apoptosis, an MTT assay was used to detect the effects of Z-IETD-FMK, Z-LEHD-FMK and Z-VAD-FMK on cell viability. The results showed that the above inhibitors had no effect on DEF viability (Figure 2B). Then, we assessed the effects of Z-VAD-FMK, Z-LEHD-FMK and Z-LEHD-FMK on viral replication. The results in Figure 2C show that Z-VAD-FMK and Z-LEHD-FMK effectively promoted viral replication, whereas Z-IETD-FMK had a weak effect on viral replication. Subsequently, the cells were pretreated with specific inhibitors of caspase-8 and caspase-9, Z-IETD-FMK and Z-LEHD-FMK, and the broad-spectrum caspase inhibitor Z-VAD-FMK and then infected with DTMUV for 36 h. Afterwards, it was determined whether Z-IETD-FMK, Z-LEHD-FMK and Z-VAD-FMK could inhibit DTMUV-induced apoptosis. We found that the specific inhibitors of caspase-8 and caspase-9, Z-IETD-FMK and Z-LEHD-FMK, and the broad-spectrum caspase inhibitor Z-VAD-FMK all inhibited DTMUV-induced apoptosis (Figures 2D and E). The above results show that DTMUV infection of DEFs promotes the activation of caspase-8 and caspase-9 cleavage and that specific inhibitors of caspase-8 or caspase-9 can inhibit apoptosis, indicating that infection activates the caspase-9-mediated mitochondrial apoptotic pathway and the caspase-8-mediated death receptor apoptotic pathway.

### DTMUV induces a decrease in MMP

**Figure 3:**
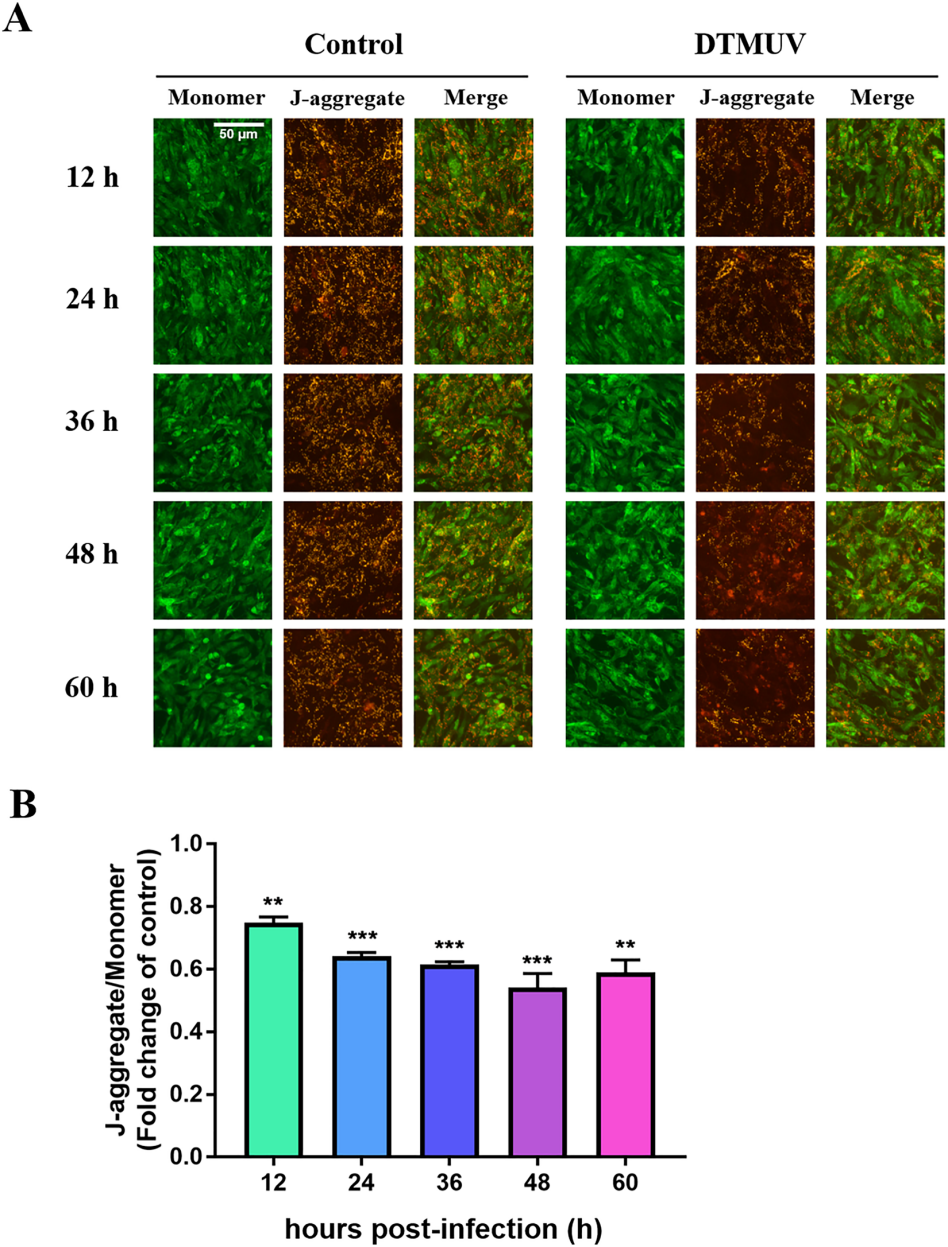
**Mitochondrial membrane potential (MMP) determination in DEFs**. **A** Assessment of DEF cell MMP through JC-10 staining and fluorescence microscopy. Mitochondria with normal membrane potential are indicated in red, and mitochondria with reduced membrane potential are indicated in green. **B** Assessment of DEF MMP with JC-10 staining and a multifunctional microplate reader. The data are presented as the means ± SEMs from three independent experiments. **p < 0.01 and ***p < 0.001, compared with the control group.

Depolarization of MMP is one of the earliest symptoms of mitochondria-mediated apoptosis [39]. Previous results have suggested that the mitochondrial pathway is involved in DTMUV-induced apoptosis. To further qualitatively confirm that DTMUV-induced DEF apoptosis occurs through the endogenous mitochondrial pathway, we used JC-10 probe technology to detect the MMP. In normal cells, in which the MMP is high, JC-1 aggregates in the mitochondria, forming polymers and emitting red fluorescence. In contrast, in apoptotic cells, in which the MMP is reduced, JC-10 cannot aggregate in the mitochondria and remains in the monomeric form, emitting green fluorescence. A decrease in MMP is a hallmark of apoptosis occurring through the mitochondrial pathway, as shown in Figure 3. It was observed by fluorescence microscopy that the red fluorescence was decreased and the green fluorescence was increased significantly at 12, 24, 36, 48 and 60 h after DTMUV infection compared to the fluorescence in the control group, which indicates that DTMUV leads to the depolarization of MMP.

### DTMUV causes accumulation of intracellular ROS

**Figure 4:**
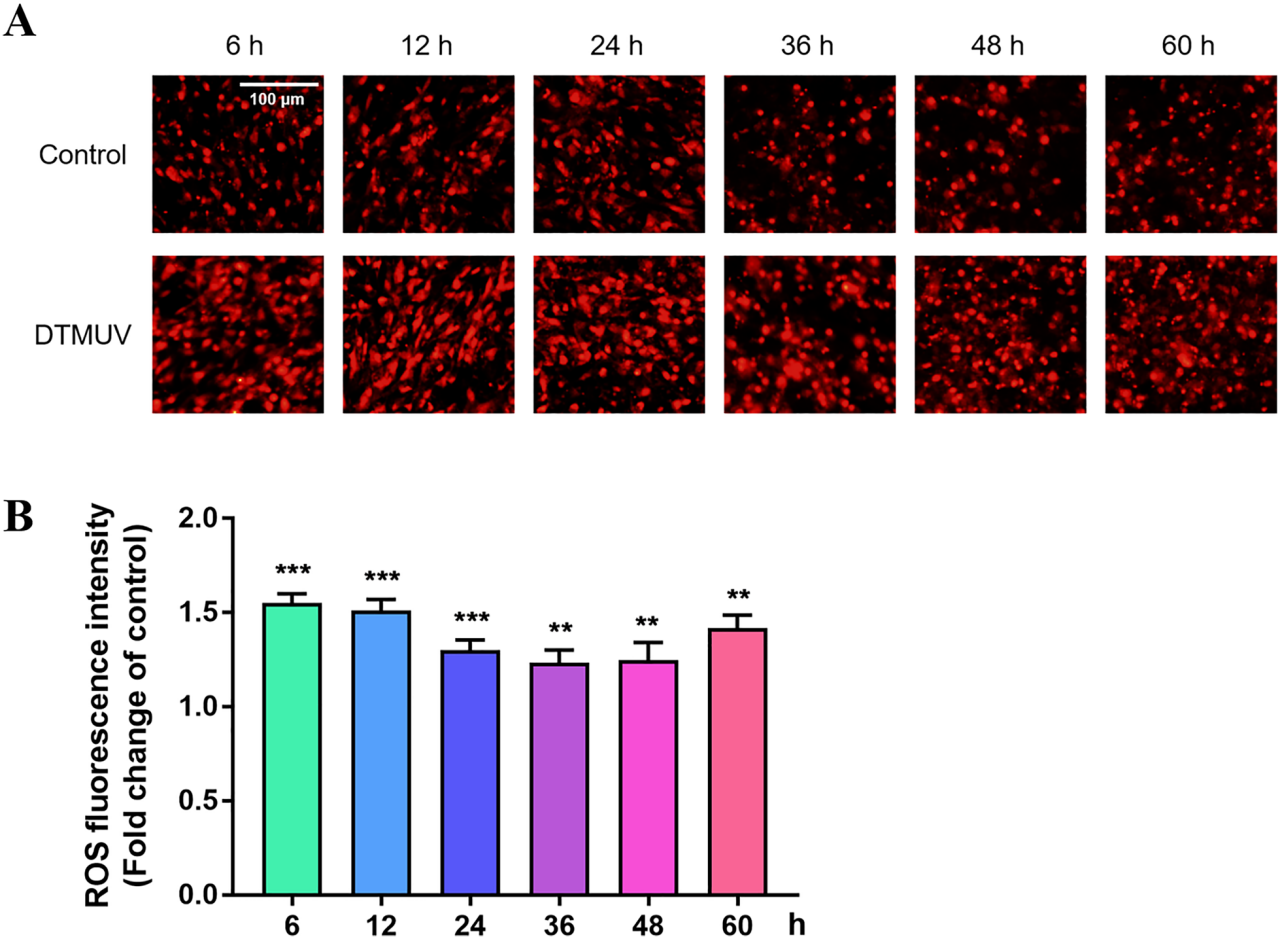
**Effects of DTMUV on intracellular ROS in DEFs. A** Assessment of ROS levels in DEFs using an intracellular ROS detection kit and fluorescence microscopy; the red colour indicates intracellular ROS. **B** Assessment of ROS levels in DEFs using an intracellular ROS detection kit and a multifunctional microplate reader. The data are presented as the means ± SEMs from three independent experiments; ** *p* < 0.01 and *** *p* < 0.001, compared with the control group.

ROS are produced by the mitochondria, and increased ROS levels reduce the MMP, which activates the mitochondrial apoptotic pathway. The results in Figure 3 show that DTMUV can lead to MMP depolarization. On this basis, we further tested whether DTMUV can cause intracellular ROS accumulation. A ROS detection kit was used to detect intracellular ROS, and the images collected by fluorescence microscopy showed that after DTMUV infection of DEFs for 6, 12, 24, 36, 48 and 60 h, the red fluorescence was enhanced compared with that of the control group (Figure 4A). The red fluorescence values detected by the microplate reader at 6, 12, 24, 36, 48 and 60 h after DTMUV infection were higher than those in the control group, and there were significant differences (Figure 4B). The results indicate that DTMUV can induce an increase in intracellular ROS and promote the occurrence of apoptosis.

### DTMUV promotes Cyt C release and inhibits Bcl-2 expression

**Figure 5:**
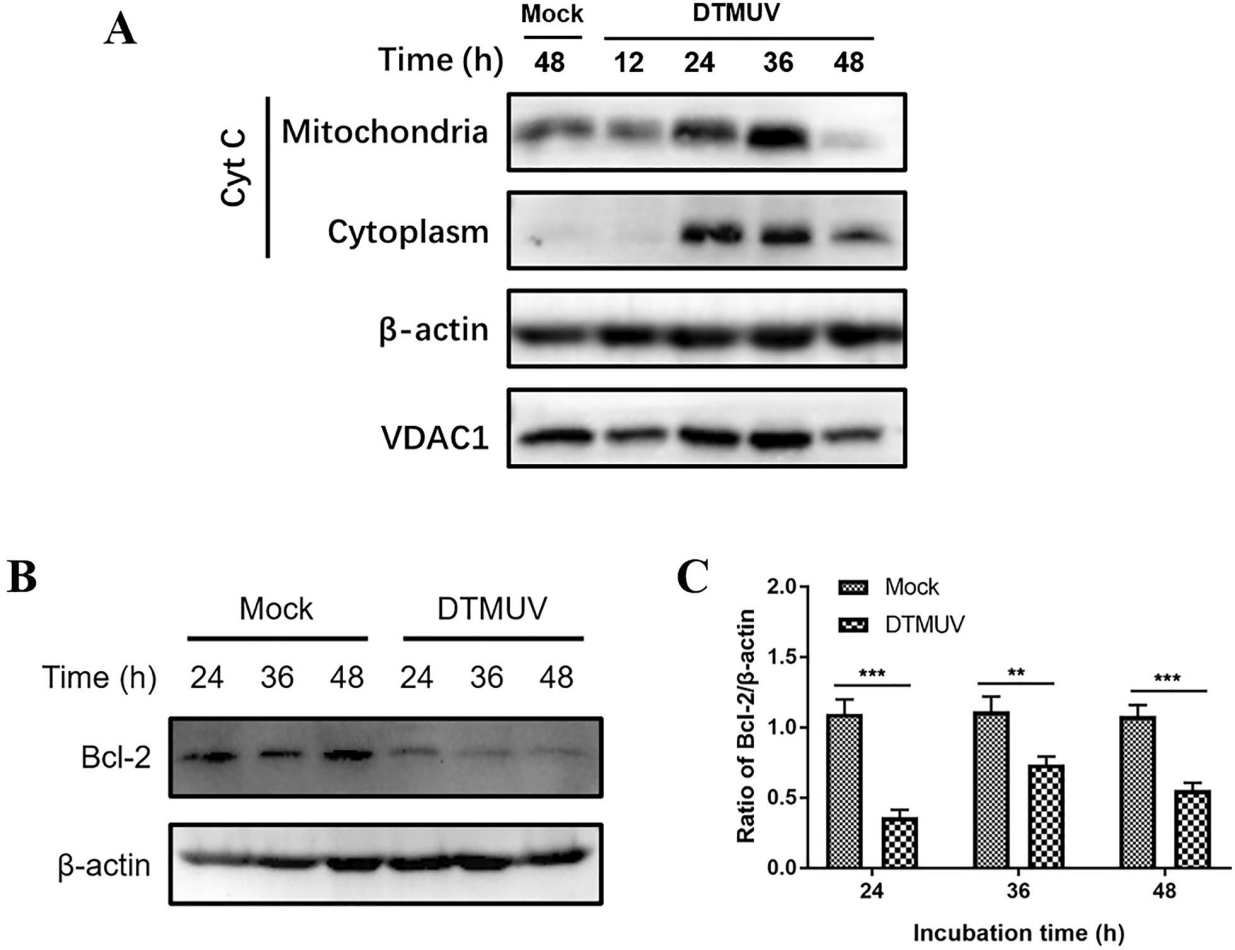
**DTMUV promotes Cyt C release and inhibits Bcl-2 expression. A** DTMUV promotes the release of Cyt C from mitochondria to the cytoplasm. **B** DTMUV infection inhibits the expression of Bcl-2. **C** The greyscale ratios of Bcl-2 to β-actin in Panel B; **p < 0.01, ***p < 0.001.

Cyt C release plays a central role in mitochondria-mediated apoptosis. The expression of Cyt C protein in the mitochondria and cytoplasm was detected by Western blotting, and DTMUV promoted the release of Cyt C from mitochondria into the cytoplasm, which confirmed that the basic structure of cell mitochondria had been destroyed (Figure 5A). The results indicate that DTMUV can induce apoptosis through the mitochondria-mediated apoptotic pathway.

The Bcl-2 protein family is an important family of regulatory proteins that regulate mitochondrial apoptosis, and the Bcl-2 protein has an antiapoptotic function. Therefore, this study continued to detect the expression of Bcl-2, and the results showed that the expression of Bcl-2 protein was significantly decreased, indicating that DTMUV infection can inhibit the expression of the antiapoptotic protein Bcl-2 to promote apoptosis (Figures 5B and C).

### Changes in the mRNA levels of key genes in the mitochondrial apoptotic pathway

**Figure 6:**
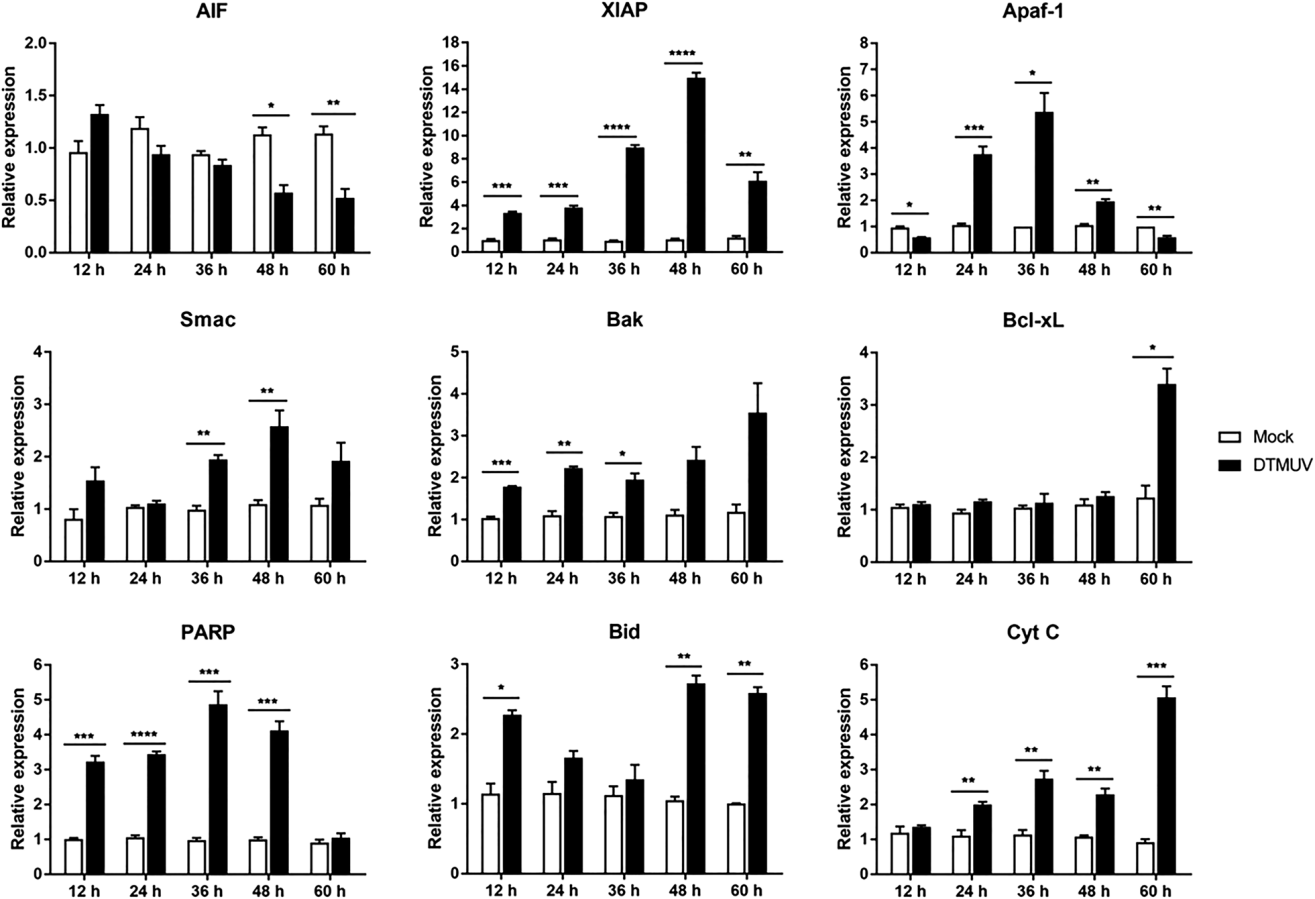
**mRNA expression levels of the mitochondrial pathway genes AIF, XIAP, Apaf-1, Smac, Bak, Bcl-xL, PARP1, Bid and Cyt C.** *p < 0.05, **p < 0.01, ***p < 0.001, ****p < 0.0001.

To determine the mRNA changes in mitochondrial apoptotic pathway-related cytokines, Q–RT–PCR was used to detect the mRNA levels of key mitochondrial pathway genes. The results showed that the mRNA levels of the proapoptotic factor Apaf-1 and the antiapoptotic protein XIAP were significantly increased at 12, 24, 36, 48 and 60 h. The mRNA levels of other proapoptotic factors were also increased: the AIF mRNA level was increased at 48 and 60 h, the Smac mRNA level was significantly increased at 36 and 48 h, and the Cyt C mRNA level was obviously increased at 24, 36, 48 and 60 h. Although the PARP protein level was downregulated, its mRNA level was obviously higher than that of the control group at 12, 24, 36 and 48 h. The mRNA levels of Bcl-2 family members (Bid, Bak, Bcl-xL) involved in the regulation of the mitochondrial apoptotic pathway were upregulated at different time points after DTMUV infection (Figure 6). The above results further confirm the activation of the mitochondrial pathway.

### Changes in the mRNA levels of key genes in the death receptor apoptotic pathway

**Figure 7:**
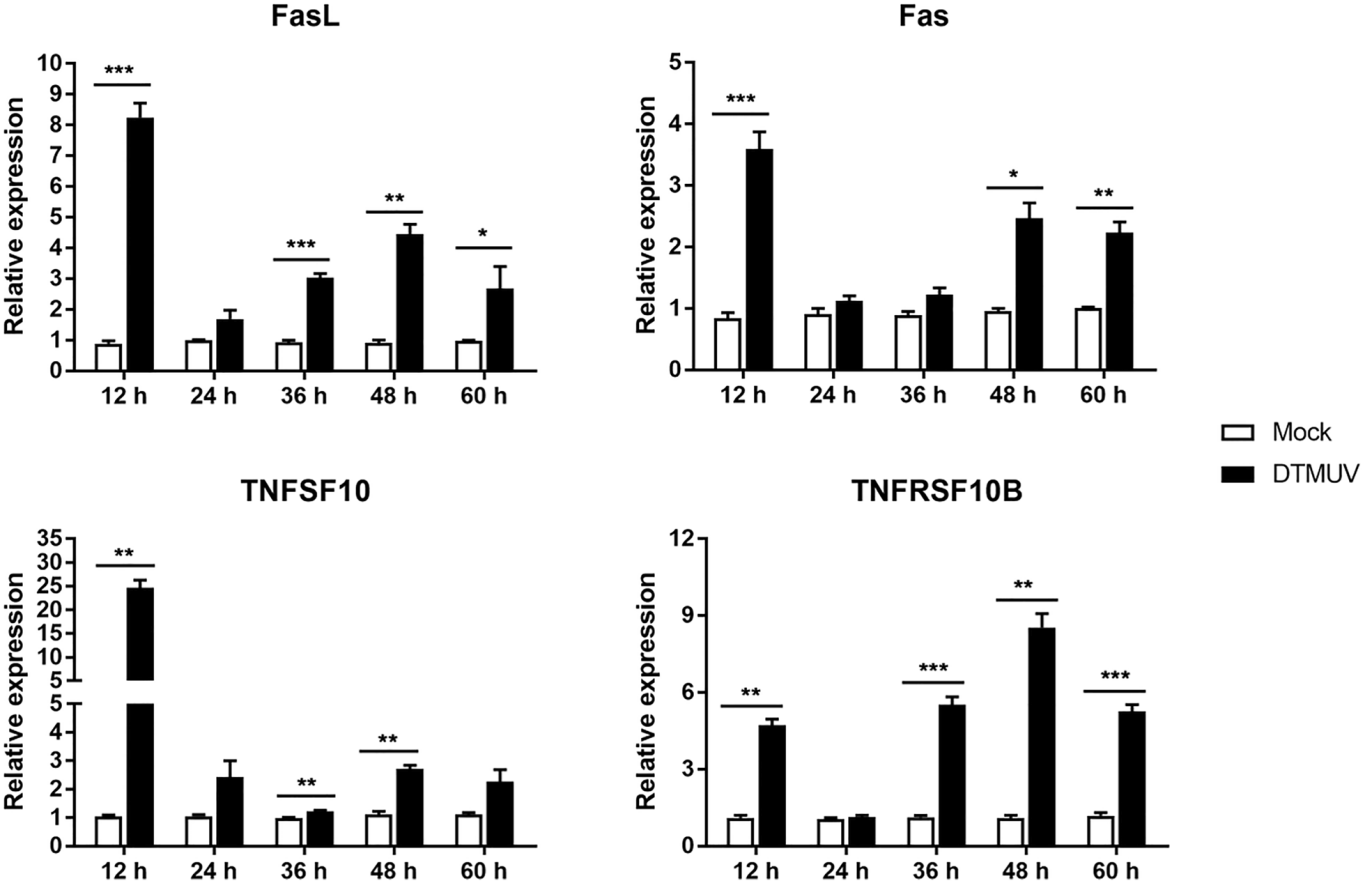
**mRNA expression levels of the death receptor pathway genes Fas, FADD, TNFSF10 and TNFRSF10B**. *p < 0.05, **p < 0.01, ***p < 0.001.

Furthermore, we also used Q–RT–PCR to detect the mRNA levels of key genes in the death receptor apoptotic pathway. Compared with those in the control group, FasL and TNFRSF10B mRNA levels were upregulated at 12, 36, 48 and 60 h. The Fas mRNA level was significantly increased at 12, 48 and 60 h, and the TNFSF10 mRNA level was obviously increased at 12, 36 and 48 h compared with that in the control group (Figure 7), which further confirms the activation of the death receptor apoptotic pathway.

## Discussion

Previous research by our group has shown that DTMUV infection can induce the apoptosis of DEFs [9], and DEF apoptosis induced by virus infection is typically time-dependent, but the signalling pathway of DTMUV-induced DEF apoptosis has not been thoroughly investigated. In this study, the cytopathic changes of DEFs were first observed by fluorescence microscopy, and it was found that virus infection caused the typical morphological changes of apoptosis, such as shrinkage of DEFs, pyknosis of the nucleus, and rupture of the nucleus. In addition, a TUNEL assay showed that DTMUV infection induced DNA fragmentation. The above results verify that DTMUV infection can induce significant apoptosis in DEFs. The classical apoptosis pathways can be divided into the mitochondria-mediated apoptotic pathway and the death receptor-mediated apoptotic pathway [40]. Caspases play important roles in the process of apoptosis. Caspase-9 and caspase-8 are involved in the mitochondrial apoptotic pathway and death receptor apoptotic pathway, respectively [41]. They can activate the downstream protein caspase-3/7 to execute apoptosis [42]. In this study, we found that DTMUV can activate caspase-3, caspase-7, caspase-8 and caspase-9. We treated cells with specific inhibitors of caspase-8 and caspase-9 (Z-IETD-FMK and Z-LEHD-FMK, respectively) and a broad-spectrum caspase inhibitor (Z-VAD-FMK). We found that the specific inhibitors of caspase-8 and caspase-9 and the broad-spectrum caspase inhibitor all inhibited DTMUV-induced apoptosis. This indicated that the caspase-8-mediated death receptor apoptotic pathway and the caspase-9-mediated mitochondrial apoptotic pathway were involved in DTMUV-induced apoptosis. Moreover, we found that virus titres were upregulated after Z-VAD-FMK and Z-LEHD-FMK pretreatment of cells, suggesting that inhibition of apoptosis contributes to DTMUV replication.

Mitochondria are organelles with a double-membrane structure. The outer membrane is more permeable than the inner membrane, allowing molecules or ions less than 5 kDa to pass through [43]. The difference in permeability between the outer and inner mitochondrial membranes is a key factor in maintaining MMP [44]. The mitochondrial apoptotic pathway is mainly caused by regulation of the mitochondrial release of Cyt C [45]. Cyt C released from mitochondria binds to Apaf-1 to activate caspase-9 and then activate downstream caspase3/7 [46]; this pathway is usually accompanied by changes in MMP [47]. The accumulation of intracellular ROS can activate mitochondrial permeability transition pore (MPTP) opening and MMP depolarization [48], while MPTP opening and MMP decrease can lead to oxidative phosphorylation and deoxygenation on mitochondria, resulting in blockade of ATP synthesis and a sharp increase in intracellular ROS [49]. Infection of porcine testicular cells (ST cells) with PDCoV results in the opening of the MPTP, the depolarization of MMP and the release of Cyt C from mitochondria into the cytoplasm, inducing apoptosis [50]. After confirming that DTMUV can activate caspase-9, we found that DTMUV infection of DEFs can promote the release of Cyt C from mitochondria to the cytoplasm and can also inhibit the expression of the antiapoptotic protein Bcl-2. In addition, according to the JC-10 probe detection results, DTMUV infection can cause the host MMP to decrease. The reduction in MMP helps to release apoptosis-related substances such as Cyt C, Smac, and AIF in mitochondria into the cytoplasm to ultimately induce apoptosis [51]. This finding indicates that DTMUV can induce apoptosis through the mitochondrial apoptotic pathway.

ROS are free radicals mainly produced by mitochondria, including superoxide anions, hydrogen peroxide, hydroxyl radicals, etc. Studies have found that ROS are involved in important biological processes, such as cell proliferation, apoptosis and autophagy [52]. Under normal circumstances, ROS are in a dynamic equilibrium. If cells are stimulated, the increase in ROS content will lead to intracellular oxidative stress, which can directly attack the MMP and trigger the release of Cyt C from mitochondria [53]. ROS can also directly affect macromolecules such as cellular DNA, resulting in DNA breakage and apoptosis [54]. This study shows that DTMUV infection can promote the accumulation of intracellular ROS, but the exact mechanism by which DTMUV regulates the accumulation of ROS is still unclear; it may involve the regulation of mitochondrial ROS metabolism enzymes. In addition, Q–RT–PCR detection showed that the mRNA levels of key factors of the mitochondrial apoptotic pathway and death receptor apoptotic pathway were upregulated to varying degrees after DTMUV infection. The above results indicate that DTMUV infection of host cells induces apoptosis through the mitochondria-mediated apoptotic pathway and death receptor-mediated apoptotic pathway, revealing the molecular mechanism of DTMUV infection-induced host cell apoptosis.

DTMUV infection triggers typical lesions of apoptosis, and caspase-8 and caspase-9 are involved in DTMUV-induced apoptosis. DTMUV activates the mitochondria-mediated apoptotic pathway by triggering the release of mitochondrial Cyt C, inhibiting the expression of the antiapoptotic protein Bcl-2, decreasing MMP and inducing intracellular ROS accumulation. In addition, the expression levels of key factors in the mitochondrial apoptotic pathway and death receptor apoptotic pathway are upregulated to varying degrees, indicating the activation of the mitochondrial apoptotic pathway and the death receptor apoptotic pathway. This study clarifies the molecular mechanism of apoptosis induced by DTMUV and provides a theoretical basis for revealing the pathogenic mechanism of DTMUV infection.

## Abbreviations

DTMUV: Duck Tembusu virus
DEF: Duck embryo fibroblast
Cyt C: cytochrome C
MMP: mitochondrial membrane potential
ROS: reactive oxygen species
PCD: programmed cell death
ERS: endoplasmic reticulum stress
EndoG: endonuclease G
TNFR: tumour necrosis factor receptor
DR: death receptor
FADD: Fas-associated death domain
TRADD: TNF receptor-associated death domain
DISC: death-induced signalling complex
NBS: newborn bovine serum
DMEM: Dulbecco’s modified Eagle’s medium
MOI: multiplicity of infection
TUNEL: terminal deoxynucleotidyl transferase-mediated dUTP nick end-labelling
PFA: paraformaldehyde
FCM: flow cytometry
V-FITC: annexin V-fluorescein isothiocyanate
PI: propidium iodide
TdT: terminal deoxynucleotidyl transferase
PARP: poly (ADP-ribose) polymerase
MPTP: mitochondrial permeability transition pore
